# Calcium Signaling in Plant Programmed Cell Death

**DOI:** 10.3390/cells10051089

**Published:** 2021-05-02

**Authors:** Huimin Ren, Xiaohong Zhao, Wenjie Li, Jamshaid Hussain, Guoning Qi, Shenkui Liu

**Affiliations:** 1 State Key Laboratory of Subtropical Silviculture, School of Forestry and Biotechnology, Zhejiang A & F University, Hangzhou 311300, China; hmren@zafu.edu.cn (H.R.); xhzhao@stu.zafu.edu.cn (X.Z.); liwj@stu.zafu.edu.cn (W.L.); 2 Department of Biotechnology, COMSATS University Islamabad, Abbottabad Campus, University Road, Abbottabad 22060, Pakistan; jamshaidhussain@cuiatd.edu.pk

**Keywords:** programmed cell death, calcium signal, hypersensitive response, abiotic stress, development, signal crosstalk

## Abstract

Programmed cell death (PCD) is a process intended for the maintenance of cellular homeostasis by eliminating old, damaged, or unwanted cells. In plants, PCD takes place during developmental processes and in response to biotic and abiotic stresses. In contrast to the field of animal studies, PCD is not well understood in plants. Calcium (Ca^2+^) is a universal cell signaling entity and regulates numerous physiological activities across all the kingdoms of life. The cytosolic increase in Ca^2+^ is a prerequisite for the induction of PCD in plants. Although over the past years, we have witnessed significant progress in understanding the role of Ca^2+^ in the regulation of PCD, it is still unclear how the upstream stress perception leads to the Ca^2+^ elevation and how the signal is further propagated to result in the onset of PCD. In this review article, we discuss recent advancements in the field, and compare the role of Ca^2+^ signaling in PCD in biotic and abiotic stresses. Moreover, we discuss the upstream and downstream components of Ca^2+^ signaling and its crosstalk with other signaling pathways in PCD. The review is expected to provide new insights into the role of Ca^2+^ signaling in PCD and to identify gaps for future research efforts.

## 1. Introduction

Programmed cell death (PCD) is a process that plays a fundamental role in plant development and responses to biotic and abiotic stresses [1,2]. According to the differences in the expression of the conserved PCD-inducing genes, two main types of plant PCD are distinguishable; developmental PCD (dPCD) regulated by internal factors, and environmental PCD (ePCD) induced by external stimuli [3]. The basic features of PCD include protoplast and nucleus shrinkage, chromatin condensation, cleavage of DNA and vacuolization [4]. The occurrence of PCD is meant to eliminate infected cells, thus limiting the proliferation of pathogenic bacteria [5].

It is reported that calcium (Ca^2+^), a universal second messenger, is critical for PCD in plants [6]. Transient changes in cytosolic Ca^2+^ level are rapidly induced by diverse stimuli in plants [7,8]. Substantial evidence indicates that Ca^2+^ plays an important role in cell death regulation [9]. The emptying of intracellular Ca^2+^ stores and/or alteration in intracellular Ca^2+^ levels has been shown to modulate cell death in almost all cell types. Ca^2+^ permeable channels and Ca^2+^ sensor CaM, CBL-CIPK and CDPK are involved in Ca^2+^ signal transduction and PCD.

## 2. The role of Ca^2+^ in PCD

### 2.1. Biotic Stresses

Plants are constantly challenged by various pathogens like viruses, bacteria, and fungi. To inhibit the spread and restrict the growth of pathogens, rapid PCD takes place at the initial infection site. Two innate immune systems play a fundamental role in PCD; PTI (pathogen-associated molecular pattern (PAMP)-triggered immunity) and ETI (effector-triggered immunity) [10,11], with the former getting more focus and hence has been better explored. The classic example of plant PCD is the hypersensitive response (HR) [12,13,14]. It is now well established that the Ca^2+^ signal is indispensable for the induction of HR. In soybean and tobacco, HR was prevented by Ca^2+^ channel blocker La^3+^ or EGTA, showing that Ca^2+^ was necessary for the induction of HR. Similarly, in *Arabidopsis*, *Pseudomonas syringae*-induced HR was preceded by an increases in cytosolic Ca^2+^, and was blocked by LaCl_3_ [15]. During the reciprocal evolution of gene-for-gene interactions, the plant’s resistance (*R*) gene product function as a signalling adaptor for the pathogen’s avirulence (*avr*) gene product, leading to refinement of HR. A study focusing on the early events in HR observed a sustained Ca^2+^ elevation downstream of the avrRpm1/RPM1 gene-for-gene interaction in *Arabidopsis* challenged by *Pseudomonas syringae pv. tomato* [16,17,18]. Overall, these studies illustrate that the Ca^2+^ signal is one of the prerequisites for the induction of HR in plants.

After the perception of different biotic and abiotic stimuli, spatial and temporal changes in cytosolic free Ca^2+^ concentrations ([Ca^2+^]_cyt_) are frequently observed as an immediate response [19,20]. The stress-induced increases in cytosolic Ca^2+^ is mediated by Ca^2+^ transporters, such as cyclic nucleotide gated channels (CNGCs), two-pore Ca^2+^ channels (TPCs), Ca^2+^-ATPases and glutamate receptors (GLRs) [21].

CNGCs mediate Ca^2+^ influx and generate the Ca^2+^ signal, which play a fundamental role in HR induced by pathogens. It was found that CNGC2 (also called DND1), is required for the induction of HR in *Arabidopsis*. cAMP-and cGMP-dependent Ca^2+^ elevation and induction of HR were impaired in *cngc2* loss-of-function mutant (also known as *dnd1*) [22,23]. CNGC4 is also implicated in pathogen defense; loss-of-function mutant of *AtCNGC4* (*dnd2*/*hlm1*) showed remarkably similar autoimmune phenotypes to *dnd1*, including defects in HR [24,25,26]. Moreover, heteropolymerization of CNGC2 and CNGC4 is necessary for the pathogen-induced intracellular Ca^2+^ influx. Loss of function of both CNGC2 and CNGC4 disrupts the downstream Ca^2+^-dependent pathogen signaling leading to HR [27]. Two other CNGC channels AtCNGC11 and AtCNGC12 also play a significant role in plant PCD by mediating Ca^2+^ fluxes [28,29]. Using electrophysiology, Zhang (2019) showed that CNGC12, but not CNGC11, is an active Ca^2+^-permeable channel in *Xenopus oocytes*. CNGC11 and CNGC12 knockout mutant plants exhibited partially decreased resistance to an avirulent oomycete pathogen *Hyaloperonospora parasitica* as well as the bacterial pathogen *Pseudomonas syringae* [30,31,32]. Interestingly, a 3 kb deletion across *AtCNGC11* and *AtCNGC12* resulted in a novel, but functional chimeric AtCNGC11/12. The mutant, named constitutive expresser of PR genes 22 (*cpr22*), exhibited increased resistance to pathogen infection in the hemizygous state and conditional lethality in the homozygous state [32,33]. Furthermore, HR-like spontaneous lesion formation in *cpr22* was shown to be Ca^2+^-dependent [34]. Moreover, Ca^2+^ channel blockers Gd^3+^ and La^3+^ suppressed AtCNGC11/12-induced PCD. Overall, these results shed light on the critical role of CNGC11 and CNGC12 in PCD. Furthermore CNGC20, a hyperpolarization-activated Ca^2+^ permeable channel, regulates *bak1*/*serk4* cell death. Notably, CNGC19, the closest homolog of CNGC20, makes a quantitative genetic contribution to *bak1*/*serk4* cell death only in the absence of CNGC20 in *Arabidopsis* [35]. As 20 CNGC members have been reported in *Arabidopsis*, other CNGCs might also be possibly involved in the regulation of PCD in plants. In addition, the heterologous combination of CNGCs increases and enriches the regulation of PCD in plants.

Besides CNGCs, other Ca^2+^ transporters also play key roles in controlling intracellular Ca^2+^ during HR triggered by pathogens. It has been demonstrated that tonoplast-localized Ca^2+^ pumps ACA4/ACA11 are main players in regulating Ca^2+^ spike induced by bacterial elicitor peptide flg22. The double-knockout *aca4/11* mutants exhibited higher basal Ca^2+^ levels as well as amplitude of Ca^2+^ signal than wild-type. These data demonstrate the important role of tonoplast-localized Ca^2+^ pumps in maintaining Ca^2+^ at homeostatic levels and for the initiation of proper PTI responses [36]. Similarly, Boursiac et al. (2010) discovered that silencing the expression of two vacuolar-localized Ca^2+^-ATPases resulted in spontaneous HR-like lesions and a faster pathogen response in *Arabidopsis thaliana* [37]. The overexpression of a rice putative voltage-gated Ca^2+^ permeable channel, *OsTPC1*, resulted in hypersensitivity to the *Trichoderma viride* xylanase (TvX) elicitor, with downstream events including oxidative burst, activation of OsMPK2, and hypersensitive cell death. On the other hand, these events were severely impaired in the insertional mutant, suggesting that OsTPC1 determines sensitivity to the elicitor and is a key regulator of hypersensitive cell death [38]. Glutamate receptors (GLRs) are also important transporters involved in mediating HR-induced intracellular Ca^2+^ influx. The increase of intracellular Ca^2+^, induced by HR, was impaired in the *glr2.7*/*2.8*/*2.9* triple mutant, which exhibited sensitivity to pathogens. These data indicate that GLR2.7/2.8/2.9 play an important role in PTI [39].

The endoplasmic reticulum (ER) stress-induced PCD is an important response pathway in plant HR. Ca^2+^ pumps on the ER membrane play an important role in this process. During the bacterial blight of rice, XA10, a kind of endogenous inducer of PCD, inhibits the ER-Ca^2+^, leading to the production of ROS in the chloroplast, and eventually leading to cell death. In addition, CPA, a specific blocker of plant ER-type IIA Ca^2+^ pumps (SERCA), can induce ER stress, and via an increase in cytosolic Ca^2+^ concentrations, triggers PCD in soybean cells. At the same time, mitochondria release cytochrome c and caspase-like activities and thereby promote PCD together [40]. Silencing ER-localized type IIB Ca^2+^-ATPase (*NbCA1*) can induce a similar extent of PCD to that induced by pathogens [41]. The evidence shows that cell death suppressor Bax inhibitor-1 (BI-1) interacts with CaM and then coordinates with Ca^2+^-ATPase to influence the ion homeostasis in plant cell death regulation [42].

In recent years some progress has been made in understanding the mechanism for regulation of these calcium transporters in HR. Cyclic nucleotides, cAMP/cGMP, can bind on and activate PM channels which mediate the flux of extracellular Ca^2+^ and increase cytosolic Ca^2+^ [43,44]. The cAMP-and cGMP-dependent Ca^2+^ elevation and induction of HR were impaired in *cngc2*, indicating that CNGC2 is a typical cAMP/cGMP dependent Ca^2+^ channel. In addition, CNGC2 is also activated by endogenous plant elicitor peptides (PEPs), leading to cytosolic Ca^2+^ elevation. Physical damage to the cells results in Ca^2+^ elevation leading to the activation of METACASPASE4 (MC4) which in turn releases Pep1 from its protein precursor, precursor of peptide 1 (PROPEP1). The released Pep1 then binds to Pep receptors (PEPRs), which activate a cyclic GMP (cGMP)-dependent CNGC2, leading to pathogen-associated cytosolic Ca^2+^ elevation to regulate HR under DAMPs in PTI. cAMP and cGMP induced Ca^2+^ signal also regulates the Pep-dependent gene expression in *Arabidopsis thaliana* [45,46,47]. CNGC11 and CNGC12 are reported to be involved in PCD. Using electrophysiology, it was shown that CNGC12, but not CNGC11, functions as an active calcium channel. Furthermore, in *Xenopus oocytes* the cyclic nucleotide monophosphates did not modulate the activities of both CNGCs. However, the activity of CNGC12 (but not CNGC11) was significantly enhanced when CaM1 was co-expressed in *oocytes* [30].

LRR receptor kinase BAK1 is located on the plasma membrane, and together with FLS2/EFR forms a complex to perceive flg22, which may involve in the initial PTI-induced cytosolic Ca^2+^ through phosphorylation, consequently negatively regulates HR [48,49,50]. Further, BAK1 interacts with and phosphorylates CNGC20 which in turn regulates CNGC20 stability. BIK1, a key component downstream of BAK1 in plant immunity [51], activates CNGC2 and CNGC4 by phosphorylation, leading to an increase in cytosolic Ca^2+^ in *Arabidopsis thaliana* [27]. Cytosolic Ca^2+^ can trigger the proteolytic cleavage of BAK1 thus negatively regulating the HR. All these studies indicate that BAK1 plays a negative role in HR induced by pathogens. However, it was also discovered that overexpression of *BAK1*-triggered cell death was dependent on SOBIR1 in *Arabidopsis thaliana* [52]. Moreover, BAK1-interacting receptor kinase 1 (BIR1) was demonstrated to be involved in the negative regulation of cell death. When the function of BIR1 is compromised, BAK1 and SOBIR1 associate with each other in plants [53]. These findings suggest that maintaining the homeostasis of BAK1 through a Ca^2+^ dependent proteolytic process is crucial for plant HR.

The stimulus-induced Ca^2+^ elevation is decoded by downstream Ca^2+^ sensors which include CaM/CMLs, CBLs-CIPKs and CDPKs. A CaM binding protein, AtBAG6, is upregulated by stress and is involved in plant PCD. The overexpression of *AtBAG6* induced the cell death phenotype in plants, which was consistent with PCD [54]. In tomatoes, the downregulation of the expression of the *APR134* gene, encoding for a CaM-related protein, compromised the plant’s immune response. Similarly, increasing the expression of *CML43* (an orthologue of APR134 in *Arabidopsis*) led to accelerated HR induced by avirulent pathogen [55,56]. These results highlight the role of the CaM-related proteins as important mediators in Ca^2+^-dependent signals during the plant immune responses. The extent of Ca^2+^ signal, ROS accumulation and PCD were significantly higher in the sensitive *Brassica oleracea* group than in the resistant group after inoculation with *Sclerotinia sclerotiorum*. Moreover, the expression of cell death-related *WRKY* transcription factors was also different between the sensitive and resistant *B. oleracea.* These findings highlight the role of WRKY transcription factors in linking the Ca^2+^ signal to downstream cell death in the host in response to *S. sclerotiorum* [57]. The calcium-dependent kinase 3 (CPK3) has been demonstrated to be a positive regulator of PCD in plants. Sphingosine or phytosphingosine (PHS) activate CPK3 which phosphorylates its binding partner, the 14-3-3 proteins. This binding leads to the disruption of the CPK3-14-3-3 protein complex and CPK3 degradation. Moreover, *Arabidopsis CPK3* knockouts exhibited the FB1-resistant phenotype, revealing a novel role for CPK3 as a positive regulator of plant PCD [58]. Recently, root meristem growth factor 7 (RGF7), perceived by the RGI4/RGI5-BAK1/SERK4 receptor complexes, acts as a novel DAMP and takes an important part in *Arabidopsis thaliana* immunity. The expression of *RGF7* precursor-encoding gene (*preRGF7*) is highly induced by *Pseudomonas syringae*, and is regulated by a signaling complex comprising of MPK3/MPK6-CPK5/CPK6-WRKY33, with MPKs and CPKs working upstream of WRKY33 [59]. It has been shown that CBL10 and CIPK6 are required for PCD triggered by kinase Pto upon recognition of *Pseudomonas syringae* effectors AvrPto or AvrPtoB in tomatoes. Ca^2+^-CBL10/CIPK6 complex promotes the accumulation of ROS by activating RbohB, and hence regulates the process of effector-triggered immunity [60]. Besides that, a study by Yang et al., (2007) has shown that *BAP* genes act as general negative regulators of biotic and abiotic stress-induced PCD. *AtBAP1* and *AtBAP2* encode small proteins containing a Ca^2+^-dependent phospholipid-binding C2 domain and interact with their functional partner BON1. The loss of BAP2 function results in promoting HR, while double mutant of *bap1 bap2* lead to seedling lethality mediated by PAD4 and EDS1, two regulators of defense responses and cell death. On the other hand, overexpression of *BAP1* or *BAP2* with their partner *BON1* abolishes pathogen-induced PCD [61].

**Figure 1:**
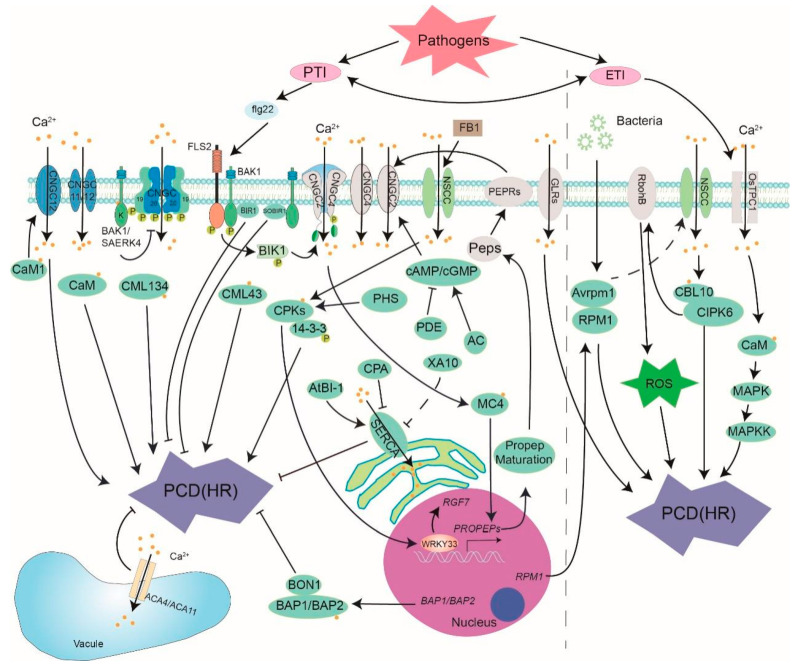
The role of calcium signal in biotic stress-induced PCD. Ca^2+^ channel, sensor and relative gene and protein are presented. PTI: pattern-triggered immunity; ETI: effector-triggered immunity; flg22: a 22 amino acid PAMP derived from bacterial flagellin; FB1: Fumonisins B1; FLS2: Flagellin-sensitive 2; CNGCs: Cyclic nucleotide gated channel; BAK1: brassinosteroid insensitive 1-associated receptor kinase 1; SERK4: Somatic embryogenesis receptor kinase 4; BIK1: botrytis-induced kinase 1; BIR1: BAK1-interacting receptor-like kinase 1; SOBIR1: suppressor of BIR1-1; Peps: plant elicitor peptide; PEPRs: extracellular Pep receptors; CaM: calmodulin; CML: CaM-like protein; CDPK(CPK): Ca^2+^-dependent protein kinase; CBL: calcineurin B-like protein; CIPK: CBL-interacting protein kinase; cAMP: 3’-5’-cyclic adenosine monophosphate; cGMP: cyclic guanosine monophosphate; AC: adenylate cyclase; PDE: phosphodiesterase; PHS: phytosphingosine; MC4: metacaspase 4; 14-3-3: 14-3-3 proteins; SERCA: sarco-endoplasmic reticulum Ca^2+^-ATPase; ACA: autoinhibited Ca^2+^-ATPase; RPM1: resistance to Pseudomonas syringae pv. Maculicola 1; AvrRpm1: Pseudomonas syringae type III effector; MAPK: Mitogen activated protein kinase (based on [10,11,12,13,14,15,16,17,18,19,20,21,22,23,24,25,26,27,28,29,30,31,32,33,34,35,36,37,38,39,40,41,42,43,44,45,46,47,48,49,50,51,52,53,54,55,56,57,58,59,60,61,62]).

Most of the previous studies in the field of plant immunity have regarded PTI and ETI as two independent parallel immunity branches, however, the latest research results show that PTI and ETI are interrelated. PTI is indispensable to ETI, plants with less efficient PTI as the first layer of the immune system also exhibit diminished plant disease resistance mediated by ETI in the second layer of the immune system. ETI can amplify PTI and induce a more lasting immunity output by enhancing the expression of core protein components in PTI, which helps plants to stimulate a strong and lasting immune response against pathogen invasion [62]. In HR-induced PCD, Ca^2+^ signals might serve as a link between PTI and ETI (Figure 1).

### 2.2. Abiotic Stress

#### 2.2.1. Salt Stress

Under salt stress, the level of reactive oxygen species (ROS) in plants like grape [63], tobacco BY-2 cells [64] and barley [65] increases and results in PCD [66]. Salt stress triggers increases in cytosolic free Ca^2+^ concentration ([Ca^2+^]_cyt_), which, as a signaling molecule, plays an important role in regulating PCD in plant cells [67]. A low concentration (10 μmol/L) of Ca^2+^ channel blocker LaCl_3_ effectively prevented the early stages of salt stress-induced PCD in rice roots by inhibiting cytoplasmic Ca^2+^ elevation and ROS production [68]. Similar to the effect of La^3+^, the overexpression of *Bcl-2*, one of the most important antiapoptotic members in mammals, significantly suppressed transient cytosolic Ca^2+^ elevations. This led to a decrease in the expression levels of *OsVPE2* and *OsVPE3* (vacuolar processing enzymes), prohibition of salt stress-induced PCD, and ultimately improved salt stress tolerance in transgenic rice [69].

Besides animals and higher plants, some physiological cell death processes (considered as a kind of PCD), have also been found in many prokaryotic microorganisms like bacteria [70] and the phytoplankton [71]. Excess Ca^2+^ can antagonize salt stress-induced cell death in prokaryotic organism *Anabaena* [72]. To date, the regulation mechanism of Ca^2+^ signal in salt stress-induced PCD is unclear. Glycosylinositol phosphorylceramide (GIPC), as a Na^+^ sensor, gates the Ca^2+^ influx channels in plants under salt stress [73]. In addition, some Ca^2+^ transporters, like annexin1 (ANN1) [74] and Ca^2+^/H^+^ antiporter (CAX1) [75], take part in the alteration of cytosolic Ca^2+^ in plants under salt stress. However, there is still no experimental evidence to demonstrate whether these components are also involved in salt stress-induced PCD.

#### 2.2.2. Temperature Stress

PCD can occur as a response to temperature stresses, including chilling and heat shock [76,77]. Under chilling/cold conditions, the transient elevation in cytosolic free calcium concentration ([Ca^2+^]_cyt_) acts as second messenger to stimulate a variety of downstream processes [78,79]. A previous study demonstrated that an alteration in the level of [Ca^2+^]_cyt_ plays a key role in regulating PCD [80]. However, the role of Ca^2+^ in temperature stress-induced PCD process is only scarcely reported. It was identified that Ca^2+^ plays an important role in the initiation and execution of cold-induced PCD in cucumber fruit [81]. To date, multiple transmembrane transport activity-related proteins, such asannexins (ANNs) and cyclic nucleotide-gated channels (CNGCs), mediating Ca^2+^ influx in response to abiotic stress, have been reported [82,83]. The G-protein regulator chilling tolerance divergence 1 (COLD1) was first established to mediate the cold-induced influx of Ca^2+^ and confer cold sensing in rice [84,85]. A previous study found that AtANN1 was involved in heat-induced [Ca^2+^]_cyt_ elevation and heat stress response [86]. A further study showed that *MYB30* negatively regulated the heat shock response partially through *ANN1* and *ANN4* [87]. Moreover, Ca^2+^-permeable transporter ANNEXIN1 (AtANN1) mediated cold-induced Ca^2+^ influx, and acted downstream of OST1 to positively regulate freezing tolerance in *Arabidopsis* [79]. In plants, CNGCs are involved in low or high temperature stress and their functions are thought to result from their involvement in Ca^2+^ influx. OsCNGC14 and OsCNGC16 play critical roles in heat as well as cold tolerance and are modulators of Ca^2+^ signals in response to temperature stress in rice [88]. Furthermore, their homologs AtCNGC2 and AtCNGC4 in *Arabidopsis* promote plant growth under chilling and improve freezing tolerance [88]. Moreover, it was reported that disruption of moss *CNGCb* and *Arabidopsis CNGC2* resulted in a hyper-thermosensitive phenotype, showing that these channels were involved in the control of the plant’s heat shock response (HSR) [89]. AtCNGC6 is a heat-activated PM Ca^2+^ channel and improves the expression of heat shock protein (HSP) genes, which enhence thermotolerance [90]. GLR3.3 and GLR3.5 were shown to mediate cold acclimation-induced chilling tolerance by regulating apoplastic H_2_O_2_ production and redox homeostasis in tomatoes [91]. Besides Ca^2+^ channels and transporters, the Ca^2+^-sensing receptor CAS has been shown to be partially involved in heat-induced chloroplast Ca^2+^ response [92]. In addition, cold and freezing can cause the change in a cell’s osmotic potential. The expression of osmotin can be activated by low temperature, and it is involved in cold acclimation-induced PCD in the olive tree and in arresting cold-induced Ca^2+^ signaling [93]. OSCA1, as an osmosensor, is responsible for [Ca^2+^]_cyt_ increases induced by water deficiency in plants. Further research is needed to explore whether OSCA1 is involved in regulating cold-induced PCD [94]. In addition to the above-described channels and transporters, membrane lipid composition can also regulate the calcium-dependent heat-signaling pathway [95]. It has been suggested that MPK6 is responsible for the activation of *Arabidopsis* vacuolar processing enzyme (γVPE) under HS stress and played an essential role in HS-induced PCD [96].

#### 2.2.3. Anoxic Stress

Plants undergo hypoxia stress under flooding. Root epidermal cells often form aerenchyma through programmed death in response to hypoxia stress [97]. Studies have shown that Ca^2+^ signaling regulates the hypoxia stress in plants. Under normal oxygen supply, both Ca^2+^ channel inhibitors and protein phosphatase inhibitors promote cell death in corn roots, while under insufficient oxygen supply, both Ca^2+^ chelator EGTA and protein kinase inhibitors prevent this process [98]. In wheat roots, hypoxia stress induced the increase in cytoplasmic Ca^2+^, which led to the Ca^2+^ accumulation in the mitochondrial matrix and the formation of mitochondrial permeability transition pores (MPTP—a factor in cell damage). These events lead to a rapid depletion of the inner membrane potential, initial contraction of the mitochondrial matrix, and release of previously accumulated Ca^2+^. All these events result in higher Ca^2+^ concentration and lead to the release of cytochrome C, and, thereby, induce PCD [99].

#### 2.2.4. Heavy Metal Stress

Heavy metals, can also induce PCD by triggering oxidative stress via the increase of ROS production [3]. Up to now, several heavy metals, including W, Ag, Cd, Al, Zn, Li, Cu, Co, Hg, Ni, Cr, Fe, have been reported to induce PCD in different types of cells of plant species [3]. Among these heavy metals, Cd is a highly ubiquitous toxic heavy metal. Because of the high physical resemblance to Cd and its importance for plant growth and development, Ca^2+^ was used to alleviate the Cd-induced toxicity [100]. Ca^2+^ is supposed to be an intracellular “second messenger” that can mediate plant responses to the biotic and abiotic stresses such as pathogen invasion, drought, salt, heat, cold and heavy metal stress [101]. Ca^2+^ signatures are perceived by Ca^2+^ sensor proteins and evoke downstream signaling responses [102]. In *Arabidopsis*, CDPKs, were found to enhance Cd tolerance through intensifying H_2_S signal [103]. Furthermore, Ca^2+^ signaling is involved in the regulation of Cd-induced cytotoxicity and cell death through the activation of the MAPK and PI3K/Akt signaling pathways [104]. A copper-tolerant species *Ulva compressa*, when *in vitro* cultivated with a sublethal concentration of copper (10 μm), showed an increase in intracellular Ca^2+^, which took place through the activation of inositol 1,4,5 triphosphate (IP_3_)-sensitive calcium channels [105,106,107]. He et al. (2017) showed that Ca^2+^ plays significant role in prohibiting the effects of NO on Al-induced PCD in peanut root tips [108]. Ca^2+^ may be involved in Pb^2+^-mediated cell death and trigger the activity of MAPK via the CDPK pathway [109]. The Ca^2+^/calmodulin system also participates in response to toxicity mediated by Pb^2+^ and Ni^2+^ [110]. It has been demonstrated that Ca^2+^ enhances tolerance against Cr stress through interacting with hydrogen sulfide in *Setaria italica*. Moreover, CDPKs are involved in Cr stress by modulating the transcriptional profiling of rice roots exposed to Cr stress [111,112]. Due to the high similarity in the ionic radii of Ca^2+^ and other cations like Cd^2+^, there is a possibility of Cd^2+^ uptake through Ca^2+^ channels or transporters. AtHMA1 functions as a Ca^2+^/heavy metal pump [113]. The mechanism of the heavy metal-mediated Ca^2+^ signature and its relationship between the Ca^2+^ signature and heavy metal-induced PCD requires in depth investigation.

#### 2.2.5. Mechanical Damage

**Figure 2:**
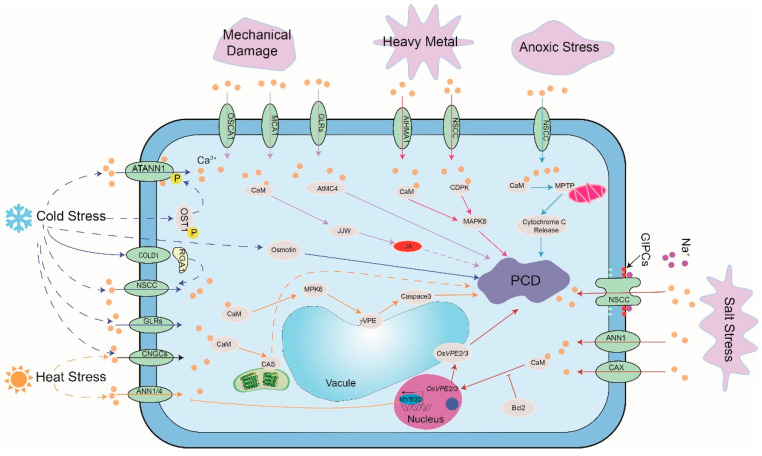
The role of calcium signal in abiotic stress-induced PCD. Salt, temperature, anoxic, heavy metal and mechanic damage stresses are depicted. OSCA1: hyperosmolality-induced [Ca^2+^](i) increase 1; MCA1: mechanosensitive channel 1; GLRs: glutamate receptor-like channels; AtHMA1: heavy metal transporting ATPase 1; NSCC: nonselective cation channel; CAX: H^+^/Ca^2+^ antiporters; COLD1: chilling-tolerance divergence 1; AtANN1: Ca^2+^- permeable transporter ANNEXIN1; OST1: open stomata 1; RGA1: rice G-protein a subunit 1; VPE: vacuole processing enzymes; JJW: JAV1-JAZ8-WRKY51 complex; JA: jasmonic acid; GIPCs: glycosyl inositol phosphoryl ceramides (based on [63,64,65,66,67,68,69,70,71,72,73,74,75,76,77,78,79,80,81,82,83,84,85,86,87,88,89,90,91,92,93,94,95,96,97,98,99,100,101,102,103,104,105,106,107,108,109,110,111,112,113,114,115]).

Plant damage due to mechanical events such insect bite and systematic wound is inevitable in nature. Plants undergo PCD in response to mechanical damage. Different proteins have been identified which link mechanical damage to downstream Ca^2+^ elevation. One such candidate is MCA1, a plasma membrane protein that correlates Ca^2+^ influx with mechanosensing in *Arabidopsis thaliana* [82]. The other candidates for the perception of injury are GLRs. Plants transform injury-induced glutamate accumulation into Ca^2+^ signals and, thereby, transmit stress signals to distant leaves mainly by GLR3.3 and GLR 3.6 [114]. In addition, hyperosmolality-gated OSCA-family channels have also been reported to be Ca^2+^ permeable channels with membrane tension activation characteristics. However, whether they participate in mechanical damage induced-PCD remains to be verified. It has been reported that CaM controls the synthesis of JA by regulating the phosphorylation of the JAV1-JAZ8-WRKY51 complex, thus controlling the plant’s response to mechanical injury [115]. Upon cellular injury, cysteine protease metacaspase4 (MC4) is instantly and spatiotemporally activated with the increase of cytosolic Ca^2+^. MC4, then, promotes the synthesis of pep1 and induces the HR and PCD [46]. Overall, these studies demonstrate that Ca^2+^ signal is important for mechanical damage-induced PCD in plants (Figure 2).

#### 2.2.6. Comparison of Ca^2+^ Signaling Components under Biotic and Abiotic Stresses

It is now well established that a Ca^2+^ signal is required for the regulation of biotic and abiotic stress-induced PCD in plants. Studies have shown that the major regulatory mechanisms between these exhibit high similarities (Table 1). Ca^2+^ elevation triggered by abiotic and biotic stimuli is mediated by the Ca^2+^ transporter on the plasma membrane and the signal is further perceived and propagated by Ca^2+^ sensors such as CaM, CPKs and CBLs. However, the sensors for perceiving abiotic and biotic stresses are different. For example, FLS2/BAK1 complex act as a pathogen receptor [49,50,51], OSCA1 as an osmosensor [94] and MOCA1 acts as a salt receptor in plant [73,116]. This is consistent with the generation of a Ca^2+^ signal in plants, for example, re-exposure to the same extent of salt stress can no longer induce Ca^2+^ signal after generating elevated Ca^2+^ under the first exposure to salt stress. On the other hand, a new Ca^2+^ signal can be induced by cold stress or exposure to flg22 [117,118,119]. This indicates that the mechanism of generating Ca^2+^ signal under various stresses varies. In addition, the genes encoding for the Ca^2+^ transporter proteins and their regulatory factors are different for plant PCDs under biotic and abiotic stresses. Therefore, it can be inferred that the process of PCD in plants is triggered by the Ca^2+^ signal acting downstream of different receptors under different stresses.

### 2.3. Plant Development and Postharvest Storage

PCD is involved in several aspects of plant growth and development, such as tissue senescence, embryogenesis, self-incompatibility, and transition from bisexual to unisexual flowers [120]. Compared with abiotic-induced PCD, the molecular mechanisms of the Ca^2+^ signal in developmental PCD (dPCD) have only partially been explored. However, a few studies have demonstrated the crucial role of Ca^2+^ in dPCD processes, such as specific tissue formation, leaf senescence and fertilization. Previous research showed that tracheary element differentiation uses a specific mechanism coordinating secondary cell wall synthesis and PCD. Moreover, through pharmacological approaches (by using either EGTA to chelate Ca^2+^ or ruthenium red to inhibit Ca^2+^ influx), it has been established that the execution of cell death requires an influx of Ca^2+^ into the cells [121]. PPF1, a putative Ca^2+^ ion carrier, inhibited PCD in apical meristems of both G2 pea (*Pisum sativum* L.) and transgenic *Arabidopsis* plants by keeping the cytoplasmic Ca^2+^ concentration at a low level [122]. Previous reports showed that an increase in Ca^2+^ concentration in the nucleus may activate the PCD in secretory cavity cells, and that Ca^2+^ elevation improved the regulation of nuclear DNA degradation [123]. Subsequently, Bai et al. (2020) found that CgCaN, a Ca^2+^-dependent DNase, directly functioned in nuclear DNA degradation during the formation of secretory cavity by PCD in *Citrus grandis* fruit [124]. More recently, it was reported that CPK1 could control senescence-related PCD by phosphorylation of senescence master regulator ORE1 [125]. In another study on senescence-related cell death, it was found that WRKY transcription factor could be phosphorylated by CPK and then CPK-WSR1 (a WRKY regulating ROS and SA) modulated two well-defined inducers of leaf senescence, salicylic acid (SA) and reactive oxygen species (ROS), to control cell death and leaf senescence [126].

Double fertilization is a unique and significant process for flowering plant reproduction. Ca^2+^ plays crucial roles in pollen tube guidance and reception. During the process, it can lead to the PCD of the pollen tube and one synergid. It has been shown that the synergid controls sperm delivery through the FER signal transduction pathway to initiate and regulate their distinct Ca^2+^ signatures in response to the Ca^2+^ dynamics and growth behavior of the pollen tube [127]. Besides involvement in double fertilization, PCD is also induced by self-incompatibility (SI) in an S-specific manner incompatible pollen, which reveals a mechanism to prevent self-fertilization [128]. In *Papaver rhoeas*, S-protein, controlling the SI, interacts with incompatible pollen and triggers a Ca^2+^-dependent signature, leading to the inhibition of pollen tube growth [129,130]. In the development of the litchi flower, researchers found that the inner integument cells of male flowers underwent the PCD, which was triggered by distributional changes in Ca^2+^ [131].

**Figure 3:**
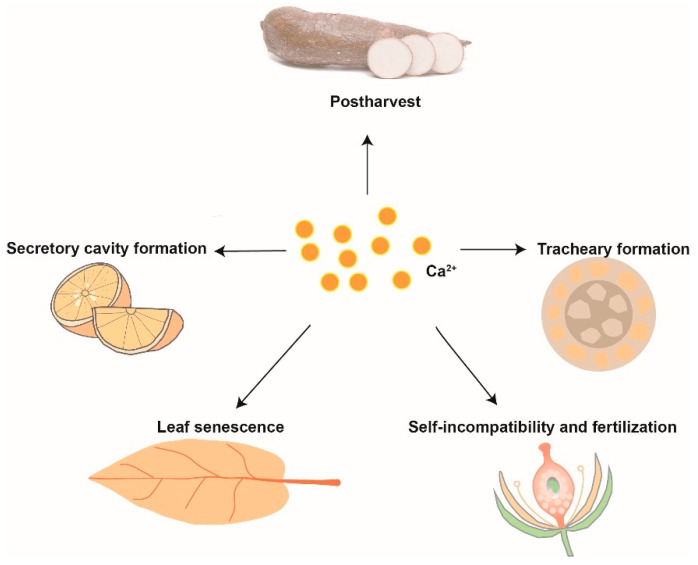
Ca^2+^ participates in the PCD during plant development and postharvest.

Postharvest physiological deterioration (PPD) of cassava storage roots is a complex process, which involves ROS, Ca^2+^ signaling transduction, and PCD [132]. Owiti et al. (2011) showed that the expression of CaM proteins was significantly upregulated, which could be the result of an oxidative burst-induced rapid increase in Ca^2+^ during early PPD. During late PPD, the PCD pathway was activated due to an increase in cysteine proteases [133] (Figure 3).

### 2.4. Small Chemical Molecule

Many chemicals can induce PCD in plants, wherein the involvement of Ca^2+^ signaling has been demonstrated. An early research report showed that Ca^2+^ plays an important role in gallic acid-induced PCD which was effectively inhibited by a Ca^2+^ chelator BAPTA-AM [134]. Thaxtomin A (TXT) is a nitrated dipeptide phytotoxin produced by all plant-pathogenic Streptomyces species, and is necessary for the realization of PCD. It has been demonstrated that TXT induces the transient Ca^2+^ increase in cells, activates the anion channel and induces the accumulation of the defense gene PAL1, until PCD takes place. Further, Ca^2+^ inhibitors La^3+^, Gd^3+^, or BAPTA inhibited the TXT-induced PCD [134], showing an important role of Ca^2+^ in this process. In addition, it has also been demonstrated that Ca^2+^ is involved in Victorin C, a host-selective cyclic peptide toxin produced by *Cochliobolus victoriae*, that induced PCD in oats [135]. Chitosan, is a component of the cell wall of many fungi and has been widely used to mimic pathogen attack. Chitosan or oligochitosan induced PCD in soybean cells and tobacco suspension cells which was suppressed by Ca^2+^ channel inhibitors [136,137]. A study has shown that endopolygalacturonase (PG), a toxin produced by *Sclerotinia sclerotiorum*, induced a rapid increase in [Ca^2+^]_cyt_ and triggered PCD in soybeans. These results were further confirmed by the observation that seedlings constitutively expressing a polygalacturonase-inhibiting protein (PGIP) did not undergo PG-induced PCD [138].

### 2.5. Metacaspases

Plant metacaspases (MCPs) are conserved cysteine proteases postulated as regulators of PCD. A study has reported that the expression of tomato type II metacaspase (*LeMCA1*) was rapidly upregulated in tomatoes during cell death induced by *Botrytis cinerea*, Similarly, in tobacco, the expression of *NbMCA1* enhanced the resistance against *Colletotrichum destructivum* [139]. On the other hand, a decrease in the expression of the type II metacaspase asperata inhibited the PCD in the suspensor cells during embryogenesis in *Picea* [140].

Nine MCPs have been reported in *Arabidopsis thaliana* [141]. The in vitro catalytic activities of recombinant type II metacaspase subfamily members AtMC4 (AtMCP2d), AtMC5 and AtMC8 were found to be Ca^2+^-dependent while recombinant AtMC9 was active under mildly acidic conditions and not dependent on stimulation by Ca^2+^ [142]. As mentioned above, AtMC4 plays a positive regulatory role in both biotic and abiotic stress-induced PCD in *Arabidopsis thaliana* [47]. The residue Lys225 of AtMC4, a highly conserved residue among the six *Arabidopsis* type II MCPs, is critical for the catalytic activation by Ca^2+^, and essential for AtMC4-mediated activation of H_2_O_2_-induced cell death in yeast [142]. The recently resolved structure of AtMC4 revealed insights into its activation mechanism. The side chain of Lys225 in the linker domain blocks the active site by sitting directly between two catalytic residues. Activation of AtMC4 by Ca^2+^ and cleavage of its physiological substrate involves multiple cleavages in the linker domain [48]. MC5 was also found to mediate defense-related PCD in tobacco [143]. Another member AtMC8 regulates oxygen stress-induced PCD in *Arabidopsis*. The expression of *AtMC8* was upregulated in UVC and H_2_O_2_ induced PCD, while the loss of *AtMC8* inhibited the cell death [144]. To sum up, these results indicate that Ca^2+^ plays an important role in MCP-mediated PCD.

### 2.6. Crosstalk between Ca^2+^ and Other Signaling Molecules in PCD

PCD is a complex biological process. Many studies on PCD in plants have shown that PCD involves an intricate network of signaling pathways, including various molecular signals, such as Ca^2+^, ROS, NO and phytohormones [145]. By regulating various aspects of cellular signal transduction in plants, Ca^2+^ plays an essential role as a second messenger. Moreover, these different signals have a crosstalk with the Ca^2+^ signal and form a regulatory network for controlling PCD in plants in response to diverse stimuli. If Ca^2+^ is increased to the level as attained just before the onset of pathogen-induced HR in soybean, PCD would not occur. This indicates that the Ca^2+^ signal needs to coordinate with other signaling pathways to regulate PCD [146].

ROS signals play an important role in both biotic and abiotic stress-induced PCD. Activated in response to Ca^2+^ signal, CDPKs subsequently activate RBOH (respiratory burst oxidase homolog) to influence ROS in different plants. Thus, RBOH acts as a hub where Ca^2+^ and ROS signaling networks crosstalk [147,148,149,150]. It was reported that H_2_O_2_ stimulates a rapid influx of Ca^2+^ into soybean cells, which triggers physiological PCD [151]. In *Arabidopsis*, a mutation in the nuclear transporter SAD2 (sensitive to ABA and drought 2) is responsible for H_2_O_2_-induced cytosolic Ca^2+^ increase. Further research showed that SAD2 works downstream of FBR11 (fumonisin B1-resistant 11) and plays a role in Ca^2+^- and H_2_O_2_-mediated cell death [6]. Recently, H_2_O_2_ sensor LRR receptor kinase HPCA1 (hydrogen peroxide-induced Ca^2+^ increase 1) has been demonstrated to mediate H_2_O_2_-induced activation of Ca^2+^ channels in guard cells [152]. H_2_O_2_ may also regulate mitochondrial permeability transition by elevation of [Ca^2+^]_cyt_. Further analysis showed that the signaling pathway for [Ca^2+^]_cyt_-mediated mitochondrial permeability transition was associated with H_2_O_2_-induced in tobacco protoplasts [153]. In *Arabidopsis*, mechanical wounding triggered the activation of MPK8 which was dependent on two factors: its direct binding with calmodulins (CaMs) in a Ca^2+^-dependent manner, and phosphorylation and activation by a MAPKK MKK3. Once activated, MPK8 negatively regulates ROS accumulation by controlling the expression of the *RbohD* gene. These results suggest that MPK8 acts as converging point for Ca^2+^ and MAP kinase pathways for regulation of ROS dynamics [144,154]. BnaCPK6L/CPK2, located at the endoplasmic reticulum membrane, interact with RbohD and regulate its activity by phosphorylation. Transient expression of BnaCPK6L or overexpression of BnaCPK2 triggers ROS accumulation and HR-like cell death in *Brassica napus* L. [12,14].

Recent evidence indicates that NO acts as an important cellular mediator in PCD and defense responses. NO mobilizes intracellular Ca^2+^, while NO synthesis depends on upstream protein phosphorylation events and cytosolic free Ca^2+^ increase [155]. In pepper, a calmodulin gene, *CaCaM1* plays important role in ROS and NO generation required for cell death and defense response [156]. In plant innate immune signaling cascades, Ca^2+^ increase and NO generation are crucial early steps and initiate HR to avirulent pathogens [22,157,158,159]. During this process, cytosolic Ca^2+^ rise could cause NO generation through CaM/CML, acting upstream of NO synthesis [22,159]. In *Arabidopsis*, CNGC2 mediates cyclic nucleotide monophosphate-dependent Ca^2+^ flux which leads to NO generation and HR. Further, the loss of function mutant of *CNGC2* (*DND1*) did not exhibit HR in response to avirulent pathogens [22].

**Figure 4:**
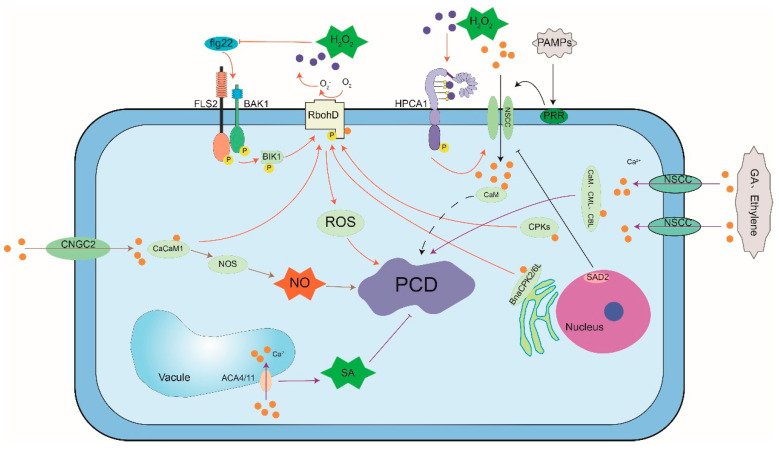
Crosstalk between calcium signal and ROS-, NO-, phytohormone-induced PCD. HPCA1: hydrogen peroxide sensor; PAMPs: pathogen associated molecular pattern; PRR: pattern recognition receptor; RBOHD: respiratory burst oxidase homolog protein; SA: salicylic acid; GA: gibberellin. (based on [145,146,147,148,149,150,151,152,153,154,155,156,157,158,159,160,161]).

Plant hormones, like SA, GA, and ethylene induce Ca^2+^ signal and play key roles in PCD. It is reported that the double disruption of Arabidopsis vacuolar pumps ACA4 and ACA11 leads to a high frequency of apoptosis-like lesions, caused during SA-dependent PCD [22,38,160]. Therefore, these vacuolar pumps establish a link between vacuolar-mediated Ca^2+^ signal and PCD in plants [38]. Okadaic acid (OA), a protein phosphatase inhibitor, can completely inhibit the GA response which is induced by rapid changes in cytosolic Ca^2+^ through regulating the gene expression and accelerated cell death [161]. Gaseous phytohormone ethylene has been reported to be involved in cell death signaling in the aerenchyma formation in the root and stems of maize (*Zea mays*) [98] (Figure 4).

## 3. Conclusions and Perspective

In this review, we focused on the role of the Ca^2+^ signal in plant PCD. In recent years, various Ca^2+^ signaling components have been identified in the regulation of plant response to diverse stresses, including the sensors of biotic and abiotic stresses. We, hereby, reviewed their link with plant PCD. However, the upstream and downstream components of these pathways remain elusive. Moreover, how the plant senses heat, mechanical damage, and heavy metal stress and how the Ca^2+^ signal is regulated and transmitted to result in PCD during these stresses need further research. In addition, the crosstalk between Ca^2+^ and other signaling pathways is not yet clear and needs further exploration. It is also not clear whether other processes for the regulation of dPCD require the input of the Ca^2+^ signal. Future studies on these research gaps are expected to broaden our understanding on the role of Ca^2+^ signaling in PCD.

## Figures and Tables

**Table 1:** The regulation factors of the calcium signal in plant PCD under biotic and abiotic stresses.

PCD		Receptor	Calcium Channel	Regulation Factor of Ca^2+^ Channel	Calcium Sensor	Substrate
Biotic stresses	PTI	FLS2/BAK1	CNGC2/4/11/12/19/20 GLR2.7/2.8/2.9 ACA4/11 SERCA	cAMP/cGMP BAK1/BIK1 PEPR	CaM/CML CPK3/5/6	RboHB 14-3-3 WRKY33 MC4
	ETI	∕	OsTPC1	∕	CaM SlCBL10	SlCIPK6 MPK
Abiotic stresses	Salt	GIPC	ANN1 CAX1	∕	CaM	OsVPE2/3
	Cold	COLD1	ANN1 SlGLR3.3/3.5 CNGC2/4 OsCNGC14/16	COLD1 OST1	CaM	Osmotin
	Heat	∕	ANN1/4 OsCNGC14/16 CAS	MYB30	CaM	MPK6 γVPE
	Anoxic	∕	∕	∕	CaM	MPTP Cytochrome C
	Heavy metal	∕	HMA1	∕	CaM CDPKs	MAPK8
	Damage	∕	GLR3.3/3.6 MCA1 OSCA1.2	∕	CaM	JJW MC4

## Data Availability

Not applicable.

## Abbreviations

PCD	Programmed Cell Death
dPCD	Developmental Programmed Cell Death
ePCD	Environmental Programmed Cell Death
CNGC	Cyclic Nucleotide-Gated Channel
CaM	Calmodulin
PPD	Postharvest Physiological Deterioration
DHS	D-Erythro-Sphinganine
MCPs	Metacaspases
PG	Polygalacturonase
MPTP	Mitochondrial Permeability Transition Pore
CBL	Calcineurin B-Like Protein
CIPK	CBL-Interacting Protein Kinase
CPK	Ca^2+^-Dependent Protein Kinase
PTI	Pattern-Triggered Immunity
ETI	Effector-Triggered Immunity
PAMP	Pathogen-Associated Molecular Pattern
HR	Hypersensitive Response
EGTA	Ethylenebis (Oxyethylenenitrilo) Tetraacetic Acid
TPCs	Two-Pore Channels
CAXs	Ca^2+^/H^+^ exchangers
cAMP	3′-5′-Cyclic Adenosine Monophosphate
cGMP	Cyclic Guanosine Monophosphate
PEPRs	Pep Receptors
DAMPs	Damage-Associated Molecular Patterns
ETH	Ecdysis Triggering Hormone
CML	CaM-Like Protein
EFR	Elongation Factor Tu Receptor
AC	Adenylate Cyclase
PDE	Phosphodiesterase
PM	Plasma Membrane
TvX	*Tichoderma Viride Xylanase*
MAPK	Mitogen-Activated Protein Kinase
BAP	Biofilm Associated Protein
SA	Salicylic Acid
RBOHB	Respiratory Burst Oxidase Homolog B
ROS	Reactive Oxygen Species
ETH	Ecdysis Triggering Hormone
GIPCs	Glycosyl Inositol Phosphorylceramides
NOS	Nitric Oxide Synthase
KEAs	Plastid K^+^ Exchange Antiporters
VPE	Vacuolar Processing Enzyme
PTP	Permeability Transition Pore
BAPTA-AM	Bis-(O-Aminophenoxy)-N,N,N,N’-Tetraacetic Acid Acetoxymethyl Ester
PGIP	Polygalacturonase-Inhibiting Protein
PG	Pyoderma Gangrenosum
HPCA1	Hydrogen Peroxide Sensor
GLR	Glutamate Receptors
PEPs	Plant Elicitor Peptides
PEPRs	Extracellular Pep Receptors
ER stress	Endoplasmic Reticulum Stress
SERCA	Er-Type Iia Ca^2+^ Pumps
PHS	Phytosphingosine
